# A comparison of the dosimetric effects of intrafraction motion on step‐and‐shoot, compensator, and helical tomotherapy‐based IMRT

**DOI:** 10.1120/jacmp.v14i3.4210

**Published:** 2013-05-06

**Authors:** Ben J. Waghorn, Robert J. Staton, Justin M. Rineer, Sanford L. Meeks, Katja M. Langen

**Affiliations:** ^1^ Department of Radiation Oncology MD Anderson Cancer Center Orlando Orlando FL USA

**Keywords:** IMRT, motion, step‐and‐shoot, compensator, tomotherapy

## Abstract

Intrafraction motion during intensity‐modulated radiation therapy can cause differences between the planned and delivered patient dose. The magnitude of these differences is dependent on a number of variables, including the treatment modality. This study was designed to compare the relative susceptibility of plans generated with three different treatment modalities to intrafraction motion. The dosimetric effects of motion were calculated using computational algorithms for seven lung tumor patients. Three delivery techniques — MLC‐based step‐and‐shoot (SNS), beam attenuating compensators, and helical tomotherapy (HT) — were investigated. In total 840 motion‐encoded dose‐volume histograms (DVHs) were calculated for various combinations of CTV margins and sinusoidal CTV motion including CTV offsets. DVH‐based metrics (e.g., $D_{95\%}$ and $D_{05\%}$) were used to score plan degradations. For all three modalities, dosimetric degradations were typically smaller than 3% if the CTV displacement was smaller than the CTV margin. For larger displacements, technique and direction‐specific sensitivities existed. While the HT plans show similar $D_{95\%}$ degradations for motion in the SI and AP directions, SNS and compensator plans showed larger $D_{95\%}$ degradations for motion in the SI direction than for motion in the AP direction. When averaged over all motion/margin combinations, compensator plans resulted in 0.9% and 0.6% smaller $D_{95\%}$ reductions compared to SNS and HT plans, respectively. These differences were statistically significant. No statistically significant differences in $D_{95\%}$ degradations were found between SNS and HT for data averaged over all margin and motion track combinations. For CTV motion that is larger than the CTV margin, the dosimetric impact on the CTV varies with treatment technique and the motion direction. For the cases presented here, the effect of motion on CTV dosimetry was statistically smaller for compensator deliveries than SNS and HT, likely due to the absence of the interplay effect which is present for the more dynamic treatment deliveries. The differences between modalities were, however, small and might not be clinically significant. As expected, margins that envelop the CTV motion provide dosimetric protection against motion for all three modalities.

PACS numbers: 87.53.Jw, 87.55.dk, 87.55.de

## I. INTRODUCTION

The dosimetric effect of respiratory target motion in intensity‐modulated radiation therapy (IMRT) plans has been studied widely using either computational techniques or direct measurement with motion platforms. However, only a few of these studies are designed to compare the dosimetric impact of motion among different treatment techniques.^1^, ^2^, ^3^ Often a choice of treatment techniques exists, so it is of practical interest to explore the relative susceptibilities of different treatment techniques to intrafraction motion. In this paper we compare the dosimetric impact of breathing motion in lung cancer patients for three different treatment modalities.

In general, dosimetric perturbations from intrafraction motion are caused by two distinct effects. The primary effect is a blurring of the dose that enlarges the dosimetric penumbra. According to Bortfeld et al.,^(4)^ the expected dose to a moving voxel can be calculated from a weighted average over the path of motion in the static dose distribution. The expected dose is independent of delivery technique, given identical static dose distributions. On the other hand, the dosimetric penumbra itself can be a function of the delivery technique and this can cause the effect of dose blurring to depend indirectly on the delivery technique. A second effect is caused by the interplay of a moving target with the treatment dynamics. This effect causes a variation of the delivered dose around the expected dose in individual fractions, and the size of this variation depends on the treatment technique.^(4)^ However, the variation from the expected value after the delivery of multiple fractions is characterized by a Gaussian distribution whose width is inversely proportional to the square root of the number of fractions.^(5)^

Studies that have compared the dosimetric effect of motion for several delivery techniques showed variations with delivery technique. An experimental study by Jiang et al.^(2)^ has shown that the mean dose error, even after the delivery of multiple fractions, can depend on the delivery mode and dose rate. Similarly, a technique‐dependent dosimetric impact of motion was reported in other experimental and computational studies.^1^, ^3^

In this paper we compare the dosimetric impact for three different treatment modalities that differ in treatment dynamic, as well as in static dose distributions. Solid compensators, step‐and‐shoot (SNS), and helical tomotherapy (HT) treatment modalities were selected since they are relatively common in the clinic but differ widely in treatment dynamics. Compensator IMRT is the least dynamic of the three delivery techniques, but each monitor unit is delivered with a spatially modulated fluence, and a moving CTV can move to regions where it is exposed to an unintended fluence. SNS treatments use mulitleaf collimators (MLCs) to deliver a multitude of static subfields, while HT uses simultaneously moving gantry, couch, and MLCs. While treatment dynamics differ among the three techniques, so do the static dose plans. HT plans use a fan beam, and treatment starts when the superior edge of the target enters the fan beam and continues until the inferior edge of the target leaves the fan beam. The dosimetric penumbra in the superior–inferior direction depends on the longitudinal extent of the fan beam, as well as the pitch, but it is in general softer compared with coplanar beam arrangements delivered with standard linacs.^6^, ^7^

The interplay effect will depend on the treatment dynamics, while the dose blurring effect widens the beam penumbra and its impact will, therefore, depend on the static dose distribution and its dose gradients. In this investigation, realistic clinical plans were used to calculate plan degradations, and sensitivities to both effects are hence accounted for. The impacts of both effects are not isolated from one another since the combined effect reflects clinical reality.

Experimental methods were used in many studies to measure variations in point doses and planar dose distributions for a variety of treatment techniques.^1^, ^2^, ^8^, ^9^, ^10^, ^11^, ^12^ However, it remains unclear how to translate these results into dose‐volume histogram (DVH)‐based parameters. The poor correlation of $D_{95\%}$ variations with gamma pass rates has recently been reported by Nelms et al.^(13)^ and Zhen et al.^(14)^ Since DVHs are typically examined during plan evaluation, it is preferable to report the dosimetric impact of motion in terms of DVH variations.

Another class of studies has used techniques to calculate and compare the impact of motion on the DVH.^3^, ^15^, ^16^, ^17^, ^18^, ^19^, ^20^, ^21^, ^22^, ^23^ Similar to the latter methods, this current study uses computational tools to report and compare the dosimetric impact of motion in terms of DVH variations. Algorithms developed by our group were used in this study.^24^, ^25^ After the calculation of a motion encoded DVH, three DVH‐based parameters, $D_{95\%}$ (the lowest dose received by the hottest 95% of the CTV), $D_{05\%}$ (the lowest dose received by the hottest 5% of the CTV), and the equivalent uniform dose (EUD),^(26)^ were used for evaluation.

The effects of motion were investigated for identical combinations of patient cases, margins, and simulated CTV movements, allowing a direct comparison between the three modalities. Sinusoidal motion tracks, with and without offsets, were used to explore the dosimetric sensitivities. This comparison should provide guidance in cases where motion is anticipated and a choice of delivery options exists.

## II. MATERIALS AND METHODS

SNS, compensator (.decimal, Sanford, FL), and HT (Accuray Inc., Sunnyvale, CA) treatment plans were created for seven lung cancer patients. The local institutional review board (IRB) approved the retrospective review of cases for the purpose of this study. The seven patients, chosen to represent a typical range of tumor sizes and locations, had physician‐contoured planning target volumes (PTVs) ranging from 135.6 to 769.5 cm^3^. For the purposes of this study, different CTV volumes were simulated for each patient by using uniform reductions of the PTV of 0, 3, 6, and 10 mm, forming four CTV and margin combinations for each plan. The clinical prescription doses and normal tissue constraints were used to create the treatment plans for each modality. The dose was prescribed to the $D_{95\%}$ DVH point (i.e., 95% of the PTV volume received at least the prescription dose). A research version of the Pinnacle treatment planning system (TPS) (Pinnacle, Version 8.1x, Philips Healthcare, Andover, MA) was used for the SNS‐and compensator‐based IMRT plans (in conjunction with the p.d version 4.2 software from .decimal), while a version of the tomotherapy planning software running on a research station was used for the HT planning. SNS plans were optimized using the DMPO (direct machine parameter optimizations) for MLC delivery. The fluence maps for each field were then exported into the p.d software for compensator design. The solid compensators were re‐imported into the Pinnacle TPS to recalculate the 3D dose distributions and to ensure clinically acceptable plans and similar DVHs to the SNS plans.

Both dose engines used a convolution/superposition algorithm. The SNS and compensator plans used a 3 mm×3 mm in‐plane resolution for the dose calculation, while the tomotherapy plans used the default calculation grid size of 128×128 voxels, equating to approximately 4 mm×4 mm. A pitch of 0.287 was used for all the HT plans, with a rotation period ranging from 15 to 20 seconds (average=16.3±2.0 seconds).

Coplanar beam arrangements of 5–11 beams were used for the SNS and compensator modalities, and a field width of 2.5 cm was used for the HT plans. Patient and planning details for each patient are listed in Table 1. A total of 28 patient‐margin combinations were investigated (7 patients×4 margins).

**Table 1:** Patient prescription and treatment plan information.

*Patient Number*	*Prescription Dose/ Number of Fractions (Gy / # Fx)*	*PTV Volume (cm* ^*3*^ *)*	*SNS & Compensator: Number of Beams*	*SNS: Average # of Segments per Beam*	*SNS: Average MU/Dose (MU/cGy)*	*Treatment Time (sec)*		
						*SNS*	*Compensator*	*HT*
1	50/20	135.58	5	9.6	1.70	266.6	194.4	288.2
2	60/30	200.46	8	8.3	2.56	414.7	321.3	257.1
3	70/35	373.31	6	11.5	2.54	339.4	242.1	274.4
4	74/37	465.31	11	6.4	3.29	401.6	277.6	405.0
5	74/37	471.48	6	10.0	2.29	322.6	235.9	231.5
6	70/35	750.01	9	9.2	3.33	493.9	362.8	420.1
7	45/30	769.52	6	7.8	3.37	350.8	225.9	418.5

### A. Motion tracks

Sinusoidal motion tracks of the form shown in Eq. (1) were used to simulate respiration‐induced CTV motion:(2,15,27,28)

(1) $$f(t)=z_{0}+A\cdot \text{sin}\left(\frac{2\pi \cdot t}{T}+\varphi \right)$$

In Eq. 1, *f* is the tumor displacement from isocenter (mm), *A* is the amplitude (half peak‐to‐peak, mm), *T* is the period of one cycle (sec), φ the starting phase (degrees), and $z_{0}$ the initial offset (mm). The initial phase correlates the beginning of the beam delivery with the motion cycle. The offset is an initial displacement of the CTV volume from its intended position (i.e., equivalent to a setup error). While a more asymmetric model can be assumed using integer powers of the sinusoidal function, it has been shown that the symmetric form of Eq. (1) provides a better fit for a number of lung tumor motions.^27^, ^28^ We, therefore, use the symmetric model in this study. The use of actual motion tracks would have been preferred, but no such data were available for the selected patients.

A total of ten different tracks were applied to each plan to analyze the effect of amplitude and offset on CTV dosimetry. Of the ten motion tracks, six had amplitudes of 5, 10, and 15 mm applied independently in the superior–inferior (SI) and anterior–posterior (AP) directions (i.e., three motion tracks of varying amplitude applied in the SI direction only and three varying amplitude motion tracks in the AP direction only). The remaining four tracks had a 7.2 mm amplitude and offsets of +3 and +5 mm in the SI and AP directions applied separately. A period of 3.8 sec and a 0° phase were used for all tracks (i.e., the start of treatment coincided with the beginning of the motion cycle). A mean respiratory period of 3.8 seconds was reported by Suh et al.^(29)^ after analyzing tracking data from over 40 patients. In clinical practice, the starting phase will vary randomly, and others have reported its impact on DVH parameters.^16^, ^17^, ^23^ The variation of the evaluated DVH parameters with starting phase was not investigated for patients since only single fractions were evaluated in this study.

The tracks were selected to test the sensitivity of each modality to different types of motion. The various combinations of margins and motion tracks resulted in scenarios where the CTV moved within, as well as partially outside of, the CTV margins. These combinations were chosen deliberately to test modality‐specific sensitivities to motion characteristics that may not be obvious in cases where the CTV moves within the margins. Since the CTV motion and CTV margins were chosen independently of each other, the PTV or ITV concepts are not used in this paper. They are, by their own definition, poor descriptors of the modeled situations. Throughout the manuscript, we use the terms CTV, motion, offset, and margin to define the modeled situations. The International Electrotechnical Commission (IEC) coordinate system was used throughout, with the patient positioned supine headfirst. Using 28 patient‐margin combinations, ten motion tracks, and three modalities, a total of 840 calculated motion‐encoded DVHs were examined for variations from the respective static plan DVH.

### B. calculation of the motion‐encoded dose distribution

The motion‐encoding dose algorithms for each treatment modality were designed around the principle of maintaining the geographical relationship between the beam's fluence and the moving CTV throughout the treatment. In effect, the beam's position relative to the patient's CT anatomy is moved to simulate CTV motion without altering the patient's CT dataset (i.e., the patient is treated as a rigid body in the algorithm). Deformation of the CTV due to CTV motion is not accounted for in any of the calculated motion‐encoded dose distributions. Using these assumptions, the motion‐encoded dose distributions can only be used to calculate the effect on tissue that moves with the same motion pattern assumed for the CTV (i.e., only CTV DVHs were calculated and analyzed). Two separate algorithms were used. The first algorithm was designed to deal with SNS‐ and compensator‐based delivery techniques. This algorithm moves the fluence of beam segments to simulate motion. A detailed description of the algorithm is presented in Waghorn et al.^(25)^ To synchronize the position of the CTV with the delivery of each MU, the following delivery timing parameters were used: dose rate=400 MU/minute for SNS and 800 MU/minute for compensator deliveries (typical clinical dose rates at our institution), intersegment interval = 1 second for SNS deliveries (estimated from sample DynaLog files storing the MLC log files for Varian treatment deliveries), and interfield beam‐off time = 40 sec for both modalities.

A second algorithm was previously developed to simulate motion for helical tomotherapy treatments. In this case, the couch position is altered for each beam projection to account for CTV motion. A detailed description of the algorithm is presented by Ngwa et al.^(24)^

### C. Intrafraction motion analysis

**Figure 1:**
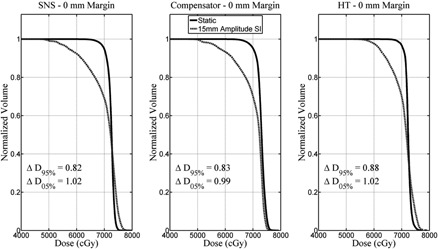
Sample static (solid line) and motion‐encoded (dashed line) DVHs for each modality, each for Patient 7 and with 15 mm amplitude SI motion applied to the zero mm margin treatment plan.

Using the 3D motion‐encoded dose distributions for each motion track, motion‐encoded DVHs were calculated and compared to the original DVHs. CTV $D_{95\%}$ and $D_{05\%}$ values were calculated for both the original (static) plan and the motion‐encoded plan. Changes between the motion‐encoded values and the static values were expressed as the quotient of the motion to static values. $\Delta D_{95\%}(=\text{Motion}‐\text{encoded}\;D_{95\%}/\text{Static}\;D_{95\%})$ and $\Delta D_{05\%}(=Motion-encoded\;D_{05\%}/Static\;D_{05\%})$ values were calculated for each motion track and each plan, where $\Delta D_{95\%}$ and $\Delta D_{05\%}$ equal unity if the motion had no dosimetric impact. Figure 1 shows sample static and motion‐encoded DVHs for each modality, each for Patient 7 and with 15 mm amplitude SI motion applied to the zero mm margin case. Additionally, the equivalent uniform dose (EUD) was calculated for each static DVH and motion‐encoded DVH, with ΔEUD being calculated as the quotient of the motion‐encoded EUD and the static EUD. A typical CTV EUD control parameter of α=−10 was used.^(26)^

Since the effect of a moving CTV is expected to depend on the size of the margin relative to the motion amplitude, all results were analyzed as a function of how far the CTV moved beyond the margin. This displacement beyond the margin was calculated by subtracting the margin (M) from the maximum displacement experienced by the CTV during the course of the fraction (i.e., ‘Max Motion ‐ Margin’ (mm)). For example, the maximum displacement of a motion track with 10 mm amplitude and no offset is 10 mm. If this track was applied to a CTV with a 5 mm margin, then ‘Max Motion ‐ Margin’ =5 mm; thus, part of the CTV moves, at most, 5 mm outside of the margin.

A statistical analysis was performed to ascertain whether any statistically significant differences were observed between the different modalities with respect to changes in CTV dosimetry following intrafraction motion. Using the absolute percent difference in $D_{95\%},D_{05\%}$, and EUD between the static and motion‐encoded DVHs, repeated measures one‐way ANOVA tests were performed across each modality. Tukey post hoc tests were performed to determine differences between subgroups. The level of significance was set at 0.05. For situations where there was a statistically significant difference between the groups, the average percent difference in the absolute deviations is reported.

## III. RESULTS

**Figure 2:**
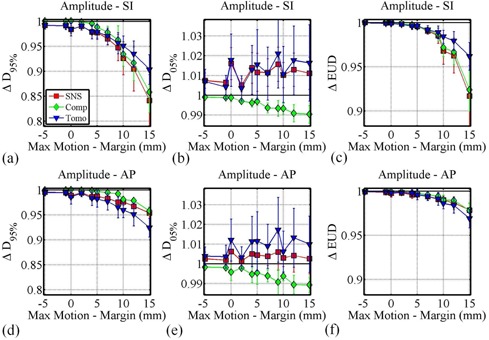
The effect of increasing the CTV displacement beyond the CTV margin (increasing ‘Max Motion ‐ Margin'), by changing only the motion amplitude and margin sizes on $\Delta D_{95\%}$ ((a) and (d)), $\Delta D_{05\%}$ ((b) and (e)), and ΔEUD ((c) and (f)) for SI ((a), (b), and (c)) and AP ((d), (e), and (f)) motion.

The effect of increasing the motion amplitude with respect to the margin is shown in Fig. 2. Data for the three dosimetric parameters are plotted for superior–inferior (Figs. 2(a)‐2(c)) and anterior–posterior (Figs. 2(d)‐2(f)) CTV motion. The x‐axis shows the maximal displacement beyond the margin, ranging from −5 mm (A=5 mm,M=10 mm) to 15 mm (A=15,M=0 mm).

From Fig. 2 it can be seen that $D_{95\%}$ is more sensitive to increasing motion relative to the planning margin than $D_{05\%}$, with EUD changes appearing to be dominated by changes in $D_{95\%}$. For all three delivery techniques, it can be observed that if the CTV moves within the margins (A−M≤0 mm), then $D_{95\%}$ degradations are typically less than 3%. For displacements of 5 mm beyond the margin, $D_{95\%}$ degradations for all three techniques were less than 5%. For larger displacements, $D_{95\%}$ degradations were more delivery‐technique dependent. While the HT plans show similar $D_{95\%}$ degradations for motion in the SI and AP directions, SNS and compensator plans displayed larger $D_{95\%}$ degradations for motion in the SI direction than for motion in the AP direction.

**Figure 3:**
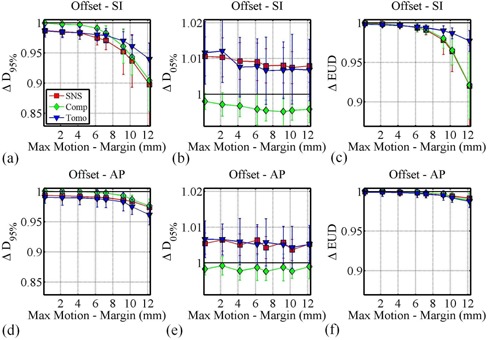
The effect of increasing the CTV displacement beyond the CTV margin (increasing ‘Max Motion ‐ Margin'), by changing only the CTV offset and margin sizes for a 7.2 mm sinusoidal motion track on $\Delta D_{95\%}$ ((a) and (d)), $\Delta D_{05\%}$ ((b) and (e)), and ΔEUD ((c) and (f)) for SI ((a), (b), and (c)) and AP ((d), (e), and (f)) motion.

The data in Fig. 3 shows the effect of adding an offset to sinusoidal motion with amplitude of 7.2 mm. The x‐axis ranges from 0.2 mm (A=7.2 mm,O=3 mm,M=10 mm) to 12.2 mm (A=7.2 mm,O=5 mm,M=0 mm). As the displacement beyond the margin increased, $\Delta D_{95\%}$ and ΔEUD decrease as expected. The respective $D_{95\%}$ degradations are similar in nature to those observed in the absence of an offset (Fig. 2).

### A. Statistical analysis

To extract differences between the dosimetric results obtained from each treatment technique, respective data were compared and examined for statistical significance. Table 2 shows results from the Tukey post hoc tests comparing the treatment modalities for a number of different motion types. The variation of the motion‐encoded $D_{95\%},D_{05\%}$, and EUD from the static dose values was scored in absolute terms looking at the absolute percent deviation from unity in the parameters; for example, $\Delta D_{95\%}$ values of 0.98 and 1.02 both exhibit the same absolute change in $D_{95\%}$ of 2%. Where there was a statistically significant difference in the mean dosimetric parameters (absolute percent variation in $D_{95\%},D_{05\%}$, or EUD) between the modalities, the modality with the smallest absolute dosimetric change is listed (S=SNS,C=compensator, and T=HT). Also shown in Table 2 is the mean difference between these parameters for the two modalities, when a statistically significant difference was observed. If the two modalities were not significantly different, N (neither) is listed.

Considering all the motion tracks used in this study (n=280 per modality), the average CTV $\Delta D_{95\%}$ for compensators was 0.98±0.03, compared to averages of 0.97±0.04 and 0.97±0.03 for SNS and HT, respectively. Using a repeated measures one‐way ANOVA test, the differences in these means were statistically significant (p<0.05), with Tukey post hoc tests showing that compensators had statistically significantly smaller changes in $D_{95\%}$ than both SNS and HT (with mean absolute differences of 0.9% and 0.6%, respectively; p<0.05). No statistically significant differences between HT and SNS plan degradations were found.

**Table 2:** A summary of the repeated measures one‐way ANOVA Tukey post hoc test statistically significant differences comparing the absolute percent deviation in $D_{95\%},D_{05\%}$, and EUD caused by motion. The modality with the least susceptibility to motion (absolute percent changes in $D_{95\%},D_{05\%}$ or EUD closest to zero) is shown (S=SNS,C=compensator,T=HT,N=neither significantly different), along with the mean difference in these values between the modalities (%) for each motion track group.

	*Compensator vs.SNS (%)*			*Compensator vs.HT (%)*			*HT vs.SNS (%>)*		
*Analysis*	$D_{95\%}$	$D_{05\%}$	*EUD*	$D_{95\%}$	$D_{05\%}$	*EUD*	$D_{95\%}$	$D_{05\%}$	*EUD*
All	C 0.9	C 0.3	N	C 0.6	C 0.6	C 0.3	N	S 0.2	T 0.4
All SI	C 1.1	C 0.6	N	N	C 0.7	C 0.8	T 1.1	N	T 0.9
All AP	C 0.6	N	N	C 1.3	C 0.4	C 0.2	S 0.7	S 0.3	N
Amplitude All	C 1.0	C 0.3	N	C 0.8	C 0.7	N	N	S 0.4	T 0.3
Amplitude SI	C 1.1	C 0.7	N	N	C 0.9	C 0.5	T 1.1	N	T 0.7
Amplitude AP	C 0.8	N	N	C 1.6	C 0.5	C 0.2	S 0.8	S 0.5	N
Offset SI	C 1.0	C 0.5	N	N	C 0.5	C 1.2	T 1.2	N	T 1.2
Offset AP	C 0.4	C 0.3	N	C 1.0	C 0.3	N	S 0.5	N	N

For the sinusoidal tracks studied here, the statistically significant advantages in $D_{95\%}$ for compensator versus SNS treatments did not translate into statistically significant EUD advantages. Some statistical differences did exist when comparing EUD changes for compensator versus HT in favor of compensators being less susceptible to motion. Differences between HT and SNS plans were also small, but HT plans had smaller changes in EUD values with SI offset and SI amplitude varying motion tracks. While some comparisons yielded differences that were statistically significant, most differences were less than 1.5% and the clinical impact of these small differences is not obvious, and may be negligible.

**Figure 4:**
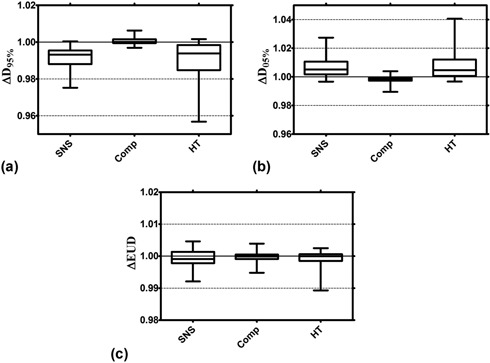
An intermodality comparison of the dosimetric effect of motion when the CTV does not move outside the margin (‘Max Motion ‐ Margin’ ≤0). The data are displayed as the median value (central horizontal line), 25th and 75th percentiles (box), and the largest/smallest values (error bars) for changes in $D_{95\%}$ (a), $D_{05\%}$ (b), and EUD (c).

Figure 4 shows an intermodality comparison of the dosimetric effect of motion when the CTV does not move outside the margin (i.e., ‘Max Motion ‐ Margin’ ≤0). Assuming that the CTV motion can be accurately predicted, this situation is inherently assumed to exist during treatment, as the PTV will envelop the CTV motion by definition. The reported data show that compensator plans exhibit smaller changes in both $D_{95\%}$ and $D_{05\%}$ than both SNS and HT plans (Figs. 4(a) and 4(b), respectively). The data in Fig. 4 are displayed as the median value (central horizontal line), 25th and 75th percentiles (box), and the largest/smallest values (error bars). The compensator $\Delta D_{95\%}$ and $\Delta D_{05\%}$ data are statistically significantly different from both the SNS and HT (p<0.05), whereas the SNS and HT data do not exhibit any statistically significant differences. Figure 4(c) shows that changes in EUD are small (less than about 1%) for all three modalities, with no statistically significant differences between the three modalities.

## IV. DISCUSSION

Intrafraction CTV motion has previously been studied widely, both experimentally and computationally. Chui et al.^(16)^ calculated $D_{95\%}$ and $D_{05\%}$ effects for lung plans delivered with dynamic MLC mode. They reported $D_{95\%}$ degradations ranging from 0.89 to 0.96 for SI PTV motion with an amplitude of 10 mm. Respective $D_{05\%}$ variations were within 1%. These results are comparable in magnitude to those reported here. For a lung‐mimicking phantom and a simple parallel‐opposed beam arrangement, an EUD degradation of less than 2% for SI motion 5 mm beyond the PTV margin was reported by Engelsman et al.^(8)^ For identical CTV motion, a EUD degradation of about 1% is reported in this study. Motion effects on HT plans were investigated by Kanagaki et al.^(21)^ using experimental methods. For a zero‐margin CTV and SI motion with amplitudes of 6 mm and 10 mm, $D_{95\%}$ degradations ranging from 0.96–1 and 0.925–0.975 were reported. Respective values from our study indicate degradations of 0.975 and 0.95. While study details may differ, this comparison with published data allows the conclusion that the absolute values reported in this study are in reasonable agreement with those of others, allowing for an intermodality comparison to be confidently performed.

Fewer studies are dedicated to the comparison of intrafraction motion sensitivities between different delivery techniques. Ehler et al.^(1)^ experimentally determined that average CTV point doses can vary by up to 9% between solid compensator and SNS deliveries for a moving CTV. However, these results were obtained for single fields that were selected for their high modulation. It is not clear how these differences translate into DVH differences. Three‐dimensional conformal, conformal arc, and intensity‐modulated radiation therapy plans were compared in terms of motion sensitivity by Wu et al.^(3)^ Results showed that of the three techniques, 3D conformal techniques are least affected by motion, while IMRT is the most affected. Contrary to the results reported in this current study, a $D_{95\%}$ degradation of 8.3% was reported in the Wu study for a CTV that moved within the PTV. In the study, Wu and colleagues used deformable image registration to accumulate dose from multiple CT phases.

Technique‐dependent sensitivities to motion are revealed in this current study. Examination of Fig. 2 shows that motion beyond the margin in the SI direction has less of an effect on HT plans compared to the other two techniques. This difference is likely due to the fact that HT dose distributions are less steep compared to the other two techniques in this direction. This dose gradient is a function of the field width. In this study, all HT plans were generated with 2.5 cm fields. Steeper or flatter dose gradients are achieved with 1 cm or 5 cm fields, respectively, with the plan's sensitivity to motion having been previously shown to vary accordingly.^(21)^ Similarly, the use of dynamic jaws as described by Sterzing et al.^(7)^ would produce steeper superior–inferior dose gradients. The sensitivity of these plans to motion will have to be reexamined.

SNS and compensator plans show less sensitivity to motion in the AP directions compared to the SI direction. Again, the steeper SI dose gradient for these coplanar beam arrangements is the likely cause. Therefore, some technique and direction‐dependent sensitivity can be explained by differences in the initial, static plan dose gradients. Static plan dose distributions are similar between compensator and SNS plans, and any motion‐induced differences between these two techniques are likely dominated by differences in the contribution of the interplay effect. Compensator plans generally showed less sensitivity to motion. While the $D_{95\%}$ differences are statistically significant, no significant EUD differences were seen. Studying the effects of noncoplanar beam arrangements and their respective changes to dose gradients in the plane of motion could provide interesting additional information, but were considered beyond the scope of this current study.

The target volumes for this study were created from a uniform reduction to the clinically determined PTV for each patient, thus simulating a margin. This approach is clearly not clinically realistic. However, for the purposes of this study where we provide direct dosimetric inter‐ and cross‐modality comparisons for varying margin sizes, the use of a single plan for each patient minimizes any variability in treatment planning that could impact the conclusions.

Drift of the target volume during treatment was not included in this study. If targets were to drift persistently during treatment, plans with a shorter treatment time would clearly be more robust against this motion. While different treatment techniques may enable a faster delivery, even identical dose distributions delivered with the same technique but at a higher dose rate would show less sensitivity to a target that drifts persistently.

The motion tracks in this study used a single sinusoidal period of 3.8 seconds. While variations in breathing frequency might cause additional dosimetric variations, particularly for the more dynamic delivery modalities, a thorough analysis was deemed beyond the scope of this current work. However, only very small changes in $\Delta D_{95\%}$ were found when comparing motion periods of 2.2, 3.8, 6.4, and 10 seconds for two SNS patients (data not shown).

As expected, margins can be used to successfully manage the effects of sinusoidal motion for all of the modalities studied here. When the margin surrounding the CTV was at least as large as the maximum motion displacement, the EUD for each modality was essentially unaffected (ΔEUD≈1). Under these situations (where ‘Max Motion ‐ Margin’ ≤0) compensators experienced a 1% smaller reduction in $D_{95\%}$, and 1% lower $\Delta D_{05\%}$ values on average than both SNS and HT treatments, likely a direct effect of differences in the contribution of the interplay effect (Fig. 4). EUD changes were again not statistically significantly different between modalities when the CTV moved within the margins.

Only the CTV dosimetry has been studied in this work as the algorithm assumes rigid body motion that is unlikely to represent the motion of the critical structures. Therefore, the conclusions made from this work only take into account the CTV dosimetry, and an analysis of the impact of motion on other structures is reserved for future work where independent organ motion and deformation can more accurately map the true patient motion.

## V. CONCLUSIONS

For all three modalities, dosimetric degradations larger than about 3% were only observed if the CTV displacement due to sinusoidal motion was larger than the CTV margin. The observed dosimetric protection from the PTV was expected for smaller target motions; however, for larger CTV displacements, technique‐specific and motion‐specific sensitivities became apparent. These differences were due to either differences in the initial static dose distributions or due to different interplay effects caused by the treatment dynamics. In these cases, careful selection of the appropriate treatment modality could reduce the dosimetric impact of intrafraction motion.

When averaged over all motion/margin combinations, sinusoidal motion, and offset in AP and SI directions, compensator plans showed smaller $D_{95\%}$ degradations of 0.9% compared to SNS plans, and 0.6% versus HT plans. These differences were statistically significant. While some comparisons in this study have shown statistically significant differences, the differences are in general small (less than 1.5%) and their clinical significance cannot be determined from this data alone.

## ACKNOWLEDGMENTS

This work was supported in part by .decimal, Inc. K.M.L. holds a research agreement with TomoTherapy/Accuray Inc.
